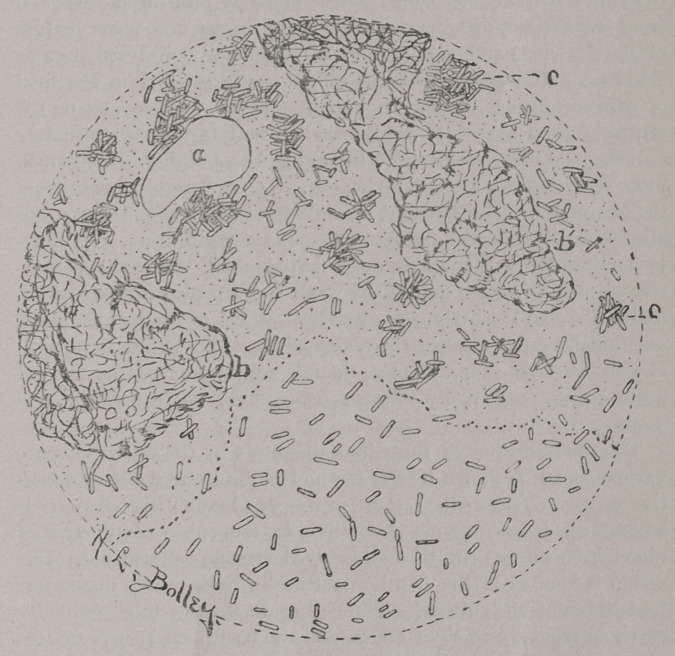# The Typhoid Serum-Diagnosis*Presented to the Section of Botany of the American Association of Agricultural Colleges and Experiment Stations, Minneapolis Meeting, July, 1897.

**Published:** 1897-09

**Authors:** H. L. Bolley

**Affiliations:** Department of Botany and Zoölogy, North Dakota Agricultural College


THE JOURNAL

OF

COMPARATIVE MEDICINE AND

VETERINARY ARCHIVES.

VOL. XVIII. *SEPTEMBER*, 1897. No. 9.

### THE TYPHOID SERUM-DIAGNOSIS.*

By H. L. Bolley, M.S.,

DEPARTMENT OF BOTANY AND ZOÖLOGY, NORTH DAKOTA AGRICULTURAL COLLEGE.

In May, 1896, Dr. R. Pfeiffer[1] wrote as follows: "In zahlreichen Arbeiter habe ich den Nachweis geführt dass in dem Blute der gegen Cholera und Typhus immunierten Menchen und Tiere spezifisches Schutzstoffe entstehen" und "Wahrend unter dem Einfluss dieser Antiköper die Cholera und Typhusbacillien im Tier organismen einer rapiden Aüflösung anheimfallen." In these statements there is indicated some of the most interesting physiological features of these minute plants. At the same time there is given a concise statement of the bases of the present theory of immunity.

During the past four years bacteriological literature is almost occupied by the various studied phases of this subject, and the amount of original investigation recorded in French and German alone is astonishingly extensive. While the separate discoveries are not flashed upon the world with the marked brilliancy and popularity of an x-ray, yet the growth is piece by piece and connectedly coördinate, and it may be doubted that any other field of work has ever, in like time, been as productive of benefits to mankind. Among the workers are Behring, Yersin, Loeffler, Gruber, Achard, Pfuhl, Kolle, Stern, Pfeiffer, and Widal. Simply a list of their labors would constitute a bibliography so long as to be tiresome. But from them all it grows each day more sure that medicine, surgery, hygiene, and sanitation are soon to be lifted upon a plane of exact science, raised there through labors more exacting and abstruse in detail than the world at large can appreciate.

* Presented to the Section of Botany of the American Association of Agricultural Colleges and Experiment Stations, Minneapolis Meeting, July, 1897.

A late acquisition of knowledge is found in the facts at the basis of the so-called serum-diagnosis. If all living bacteriologists were alone accredited with but the unearthing of the facts embodied in the two quotations from Pfeiffer, it could but be agreed that the world would be much their debtor; for, as science is superior in thought to practice, so is human life superior to all other considerations.

In July, 1896, at a meeting of the Société Médicale, Widal[2] cited a short method of diagnosing typhoid fever by observing the clearing action of blood-serum upon a living bouillon-culture of B. typhi-abdominalis. Later[3] (see *La Semaine Medicale*, October, 1896, p. 410) he simplified the method so that a rapid microscopical diagnosis became possible. This was recognized as being of the utmost importance, and the literature of the subject is already so extensive that this note, except, perhaps, for some individual features of observation, seems almost superfluous. The work, also, at first sight, seems hardly botanical, but the physiology of this parasitically inclined saphrophyte is accountable for the characteristics of the disease and for the features of immunity, and of itself is none the less within the field of plant-activity, instructive and interesting.

The Widal test is conducted in various ways, but in all cases depends upon the effects of typhoid-affected serum upon the specific bacilli; and, for those who have been fortunate enough to do comparative work upon this subject, it seems to me that the bugbear of bacteriological transformation of species must be forever relegated. It becomes evident that B. abdominalis is B. abdominalis, and B. coli communis is B. coli communis, etc. If to a living culture of either of these bacilli blood-serum is added from a patient sick of either, the reaction is specific only for the one causing the illness. In essential, the reaction consists in an almost immediate active clearing of the culture due to the paralysis and clumping "agglutination"—"zusammenballen," or "haufchenbildung" of the previously free-swimming bacilli. This may be studied in an ordinary microscopical manner by the hanging-drop method, and presents some interesting features for chemico-botanical studies, besides allowing for an instant diagnosis for the disease which doctors have always been compelled to allow to run too long upon surmises based upon supposed typical clinical features.

It is not in place to give a *résumé* of the work pro and con on the effectiveness of the test. The work of Pfeiffer and Kolle[4] is alone sufficient to show its value against all apparent exceptions. My own work has also been sufficient to allow me to feel assured that a skilled bacteriologist and microscopist need seldom, if ever, fail upon typical cases. Complications of disease are not part of this question.

ORIGINAL WORK. During the past winter and spring I have conducted studies upon four phases of this subject: first, characteristic effects of typhoid-serum upon the bacilli; second, variations in condition, as affecting the reaction; third, the use of the reaction in water-analysis; fourth, the source of reaction-serum for the last purpose.

The accompanying drawing representing a microscopic field will show at a glance the idea of a typical reaction by the dry-blood method. Every worker must find for himself the way in which he can be accurate. My method of diagnosis has been a modification of the hanging-drop method. Three drops of blood have been so placed upon the glass slip as to make raised prominences upon which the cover-glass rested, the drop of bouillon-culture

(twenty-four to forty-eight hours old) filling the remaining space; *b* and *b'* in the drawing represent patches of such blood-masses. As the active inhibitory substance within the blood-serum penetrates the liquid which surrounds the blood-blotches, as shown by the stippled area of the drawing, a peculiar influence dominates the action of each bacillus as it is seen to swim into the field of serum. Often they strike it with an apparent shock, slow up and become irregular in their course, revolve a few times about one end, as though stuck to something slimy, then lie paralyzed. Others swimming along more evenly and forcibly strike upon the still body of another bacillus, as though attracted to it, and are then stranded there like driftwood. The result is a speedy clearing of the field from free-swimming germs (compare the upper and lower halves of the drawing) and a clumping of completely paralyzed ones as seen at *c*. In case of intense reaction, bacilli swim into the field of serum-influence, stop as though by a shock, spin around on the centre of their axes with great rapidity, and then are immediately stilled. To me, though quite beyond words, nothing can be much more specific than a typical reaction, if observation is made from the beginning. The constant occurrence of the fever in Fargo has allowed me a sufficient number of cases for verification. In nineteen clinically well-marked cases first studied, eleven gave positive results, two were doubtfully weak but afterward reacted well, five failed and the early convalescence seemed to confirm the tests, one failed, though the patient remained very ill. The reaction with B. coli communis for this case, though not very emphatic, was yet quite paralyzing in its effects; hence, here was a case in real doubt.

The modifications of this direct diagnosis by the microscope are various, but it is shown by Pfeiffer and Kolle to be liable to deceptive errors. The method most in use by German investigators is accomplished by drawing from five to ten cubic centimetres of blood from the patient, then allowing the serum to settle out. The serum is applied to the bouillon-culture in bulk, using about one part to ten of the culture. The culture in the test-tube gradually clears in from six to eight hours, and the bacilli are then found to be precipitated in small clumps, "kugelchen" or "haufchen." Pfeiffer[5], however, contends that the most delicate and reliable test consists in making interabdominal injections of sero-cultural mixture. Twenty minutes after such an injection it is found that the bacilli in the interabdominal fluids have undergone "Auflosung." This is beyond the work of ordinary investigators, but is of great interest, in this connection, in the wonderful activity indicated in the rapid dissolution of the organisms.

As in the action of all natural law, the reaction is found by different observers to vary greatly according to conditions; hence many chances for error arise, and there is much opportunity for side investigations. Among the features which have come under my own observation are those of virulence and attenuation, age of the culture, liquid condition of the culture, acidity or alkalinity of the culture, and the age and condition of the blood-serum. Observed results upon these features are confirmatory of those made by many others. Each of these features, varied from what may be termed the normal results in some variation of the reaction; thus, I failed to get the reaction upon a culture direct from the spleen of a guinea-pig, except in the most marked cases, while an old culture attenuated through many transfers was found very delicate under the same conditions. The culture must, of course, be in a motile condition to give the results characteristic of the hanging-drop, inasmuch as the attendant effects upon the movement of individual germs and certain features of the clumping is dependent upon this condition. This demands a liquid culture, for the power of free swimming is developed only in such. The age of the culture is important for less obvious reasons, though loss of the characteristic motion of the free-swimming bacilli is here also the most evident and confusing feature. Bacilli from a liquid culture several days old seem also to have acquired a sort of immunity to the paralyzing action of the serum, and lie in a semi-motile condition many minutes in the strongest type of serum, complete paralysis not occurring as in the active fresh cultures. This would be easily explained could it be proved that the substance formed by them in culture is identical to that which is formed by them in the animal body. Pfeiffer,[6] however, claims that the protective substance formed is not a chemical unity, but that, at least, two different substances are formed through the direct incorporation of the body-substance of the bacilli.

I obtained negative results with cultures grown in Parietti's bouillon, potato-broth, and in other acid cultures, but positive reactions with neutral or properly alkaline bouillons. No reason is, as yet, assignable, but Cantani,[7] in conformity to the observation of many others, that immune blood is alkaline, has shown in the case of diphtheria that the alkalescence is considerably increased during the production of artificial immunity, even after the first injection. This point alone seems sufficient to cause one to believe that alkalescence is essential to the action of the protective substance against the bacilli; hence the necessity for the alkaline basis for the Widal test-reaction.

The temperature condition under which the bacilli have been grown has no effect, except that the ageing of the culture occurs more rapidly under the effects of oven-culture than in room temperature. Jes[8] has recently published similar comparative results. The germs may also be killed by heat without losing the agglutinating power, provided the temperature does not exceed 57° to 60° C. for twenty to thirty minutes. The power also persists in bacilli dead through the fumes of formalin,[9] a feature which is further indicated in the longer-known fact that proper injection of the dead bodies of bacilli results in the production of immunity.[10]

A point of great value in the diagnosis by use of the microscope is the persistence with which dried blood-serum retains the inhibitory, paralyzing, and agglutinating powers. Thus, for four weeks, I made daily use of one small, dried mass of typhoid blood. This sample retained its power to the last. Other samples less positive from the first, quite rapidly diminished in agglutinating power. I have, however, had good results from blood-drops dried upon the glass slide for three months.

A feature, which yet remains clouded only because insufficient time has elapsed since Widal's discovery, is that arising to form the question of the duration of immunity. Though not properly within the field of this paper, it may be said that the time-test may be made with certainty only upon persons who have previously given the reaction or upon those now giving positive results due to past sickness. The negative results so far reported might only indicate that clinical diagnoses of years ago were in error. However, I notice that the blood of a typical case markedly active at convalescence, six months since the convalescence seems less quick. Further, cases of long standing (six months to three years) seem slower and less clean-cut in the paralyzing and clumping processes. Widal has reported cases of seven years' standing since convalescence, and Uhlenhuth[11] records one each for periods of eight, nine, and eleven years.

The limit of reliable reaction cannot be stated by one investigator for another. Many side conditions of manipulation intervene. The limit is personal with the observer, and based upon observation, which, of course, must be obtained by comparison of many tests upon normal and pathogenic sera. For example, if my own blood is allowed sufficient time, it gives quite positive clumping. The clumping is so satisfactory that it could easily lead to error, though I am not aware of ever having had an attack of typhoid. There is, however, in this blood no proper paralysis. This last feature is, perhaps, the chief objection to the bouillon-mass test. Jes[12] has said that many normal sera affect the typhus bacillus in bouillon after the same manner described as characteristic for typhus sera. This, I think, would appear true after any but the most careful methods of work and observation, but the facts now under record are of none the less significance. Old theories of immunity have already suffered a necessary revision, and the field of physiological chemistry is more clearly open than ever.

In regard to the use of the reaction in water-analysis, there is not much experimental basis for assertion. Stoddard definitely denies that the bulk-serum test may be considered reliable for a separation of B. coli communis and B. typhi abdominalis,[13] asss erting that B. coli communis is not a well-defined species, and that some of the varieties react as does the typhus bacillus. However, it appears that he is too eager to propose a new method of separation to weigh well evidence already given by others. As his new method is nothing more than almost every working bacteriologist has probably brought into use for several years, I cannot let his argument weigh even against my own limited tests, which have been positive as far as carried. It is not supposed that any bacteriologist will rely upon a single feature of diagnosis when a dozen are at hand to make belief more certain. By the reaction for the hanging-drop method I have been able to separate typhoid bacilli from an artificial mixture of typhoid and B. coli communis and two other microscopically similar water-forms. Also, when confused in other tests in trying to differentiate between five similar cultures from sewage-contaminated river-water, this test allowed me to select the correct culture, as attested by later inoculation and post-mortem studies upon guinea-pigs.

No good worker is sure of a diagnosis so long as there is a possibility of error. So I think one need not depend upon this test alone; but, added to the other features open to present knowledge, I am quite certain this reaction furnishes the strongest test for microscopic methods. Consideration of all means available is necessary to good work, but to those prepared for inoculation and post-mortem examinations this reaction will be looked upon as but making the selection of the desired bacillus more certain prior to the final test of its character.

Serum for the hanging-drop method of fever-diagnosis is readily obtained from a slight skin-blister, pin-prick, or other slight cut, but I have found trouble to even have so much given in some cases. American patients do not seem so liberal with their blood-supply as European scientists demand. It is, however, not necessary to rely upon the human supply when it is necessary to have a quantity of the test-serum for examination of many separate cultures, though one scientifically inclined patient easily furnishes enough blood from a single pin-prick to test many slides. Guinea-pigs readily become immune through artificial protective inoculation, and the serum gives equally good results. One has only to arm himself with a sufficiently virulent culture, an injecting-syringe, and a pig, and proceed by properly graded, ever-increasing injections until blood drawn from the ear of the animal gives the desired reaction. This reaction begins to show in the blood after the first heavy illness of the animal, and becomes heightened after each recurring injection, and it is soon found that the animal is practically immune.

The reaction may be obtained by the use of the serous fluids from various organs of the body, as liver, spleen, gall, lymphatics, and other glandular structures,[14] but it is most intense in the blood-serum, which also furnishes the easiest source of supply.

In conclusion, I wish to express my thanks to Dr. Novy, of Ann Arbor, and Dr. Wesbrook, of Minneapolis, for cultures of B. abdominalis kindly furnished me by them, and, as touching further upon the specific nature of the reaction, I quote from the able investigator, Loeffler:[15] "Durch die Behandlung von Hunden mit steigenden Dosen Virulenter Kulturen der Typhusbakterien bezw. Colibakterien worden in dem Blute dieser Tiere Körper erzeugt welchen eine spezifische Schutz wirkung innewont, nur gegenueber derjenig Bakterienarten, welcher sie ihre erstehung verdanken." This, it seems to me, is in accord with the facts.

### BIBLIOGRAPHY.

1. Centralblatt für Bakteriologie und Parasitenkunde, 1896, Band xix., No. 16.
2. La Semaine Médicale, 1896, p. 250. (Cited in Centralblatt f. Bakt. und Parasitenk., Bd. xxi. p. 52.)
3. La Semaine Médicale, October, 1896, p. 410.
4. Differential Diagnose der Typhusbacillus vermittetest Serum der Gegen Typhus immunierten Tiere. Deutschen med. Wochenschrift, 1896, No. 12. (Cited from Centralblatt f. Bakt. und Parasitenk., Bd. xix., No. 24.)

Centralblatt für Bakteriologie und Parasitenkunde, 1896, Band xix., No. 16.

Ueber die spezifischer Immutatsreaktion der Typhusbacillen, Detusche med. Woch., 1894, No. 48.

Weitere Untersuchung uber die spezifischen Immunitatsreaktion der Choleravibronen in Tierkerper und Reagensglasse. Centralblatt für Bakteriologie und Parasitenkunde, Bd. xix. p. 129.

5. Loc. cit., No. 4.
6. Centralblatt für Bakteriologie und Parasitenkunde, Band xx., No. 16, p. 129.

7. Cantani: Ueber der Alkalescence des Blutes bei active immunisierten Tieren. Centralblatt für Bakteriologie und Parasitenkunde, Band xx. pp. 566–573, No. 16.
8. Ueber die Bedeutung der Widalschen Sera-diagnostik, etc. Wiener med. Blatter, 1897, No. 3 (cited from Centralblatt f. Bakteriologie u. Parasitenk., Bd. xxi., No. 16, p. 617.
9. La rèaction agglutinante sur les bacilles Mortes. La Semaine Médicale, 1896, p. 38.
10. Pfeiffer and Kolle: Experimental Untersuchung zur Frage der Schutzinepfung des Menchen gegen Typhus abdominalis. Deutsche méd. Wochenschrift, 1896, No. 46.
11. Beitrag zur Serumdiagnose bei Typhus abdominalis. Deutsche Militariarztlische Zeitschrift, 1897, Heft 3. (Cited from extract in Centralblatt f. Bak. u. Parasitenk., Band xxi. p. 698.)
12. Loc. cit.
13. New Method of Separation of the Typhoid Bacillus and B. Coli Communis, etc. Journal of Bacteriology and Pathology, June, 1897.
14. Arloing. La Semaine Médicale, 1897, pp. 38 and 69.
15. Loeffler and Abel: Ueber die spezifischen Eigenschaften der Schutzkörper in Blute Typhus and Choler iummunier Tiere. January, 1896, Centralblatt für Bakteriologie und Parasitenkunde, Bd. xix. pp. 51-70.

## Figures and Tables

**Figure f1:**